# Voltage-gated sodium channels were differentially expressed in human normal prostate, benign prostatic hyperplasia and prostate cancer cells

**DOI:** 10.3892/ol.2014.2110

**Published:** 2014-05-02

**Authors:** BIN SHAN, MEI DONG, HE TANG, NA WANG, JIN ZHANG, CHANGQING YAN, XIAOCUI JIAO, HAILIN ZHANG, CHUAN WANG

**Affiliations:** 1 Department of Pharmacology, Hebei Medical University, Shijiazhuang, Hebei 050017, P.R. China; 2 Department of Surgery, The Affiliated Hospital of Hebei Science and Technology University, Shijiazhuang, Hebei 050018, P.R. China; 3 Department of Urology, The First Hospital of Shijiazhuang, Shijiazhuang, Hebei 050011, P.R. China; 4 Department of Hepatobiliary Surgery, The Second Hospital of Hebei Medical University, Shijiazhuang, Hebei 050017, P.R. China

**Keywords:** voltage-gated sodium channel, mRNA, prostate, cancer, benign prostatic hyperplasia

## Abstract

Voltage-gated sodium channels (VGSCs) are expressed not only in excitable cells but also in numerous metastatic cells, particularly in certain types of cancer cells. In some types of cancer, including prostate cancer, the expression of VGSCs is associated with cancer migration, invasion and metastasis *in vivo*. However, the detailed expression profiles of VGSC α subunits in normal human prostate, in prostatic hyperplasia and prostatic cancer remain controversial. In the present study, quantitative polymerase chain reaction was used to systematically detect all subtypes of VGSC α subunits in normal human prostate, benign prostatic hyperplasia (BPH) and prostate cancer cells. The expression profile of VGSC α subunits was observed to differ between these cell types. Nav1.5 was the major isoform expressed in normal human prostate tissue, while Nav1.5 and Nav1.2 were the predominant isoforms in BPH tissue. However, in PC-3 and LNCaP cells, two typical prostate cancer cell lines, Nav1.6 and Nav1.7 were abundantly expressed. By comparing the relative expression levels of Nav1.5, Nav1.6 and Nav1.7 in these cells, the mRNA levels of Nav1.6 and Nav1.7 were identified to be 6- to 27-fold higher in PC-3 and LNCaP cells than in either normal or BPH samples (P<0.05); however, Nav1.5 mRNA levels were relatively lower compared with those of Nav1.6 or Nav1.7 in all cells analyzed. To confirm whether Nav1.6 and Nav1.7 expression in cancer cells was functional, a patch-clamp technique was used to record whole-cell currents. A tetrodotoxin-sensitive sodium current was successfully recorded in PC-3 cells, but not in LNCaP cells. It was concluded that although all types of VGSC α subunits exhibited low expression levels in normal prostate and BPH cells, both Nav1.6 and Nav1.7 were significantly upregulated in the prostate cancer cell lines, suggesting these subtypes may be potential diagnostic markers and therapeutic targets for certain types of prostate cancer in humans.

## Introduction

Voltage-gated sodium channels (VGSCs) are responsible for the rising phase of the action potential in the majority of electrically excitable cells and, thus, are important in impulse generation and propagation (1). VGSCs are composed of a pore-forming α subunit and one or more auxiliary subunits (β1–β4). Nine sodium channel α subunits (Nav1.1–Nav1.9), encoded by the SCN1A-SCN5A and SCN8A-SCN11A genes, have been found in vertebrates (2). Different VGSC α subunits are abundantly expressed in the majority of excitable tissues. For example, in the heart, Nav1.5 is the main subtype of sodium channel. The functions of VGSCs in such excitable tissues are well understood. VGSCs contribute to impulse generation, conduction, axonal migration and synaptic connectivity (1). Therefore, dysfunction of VGSCs leads to several diseases in excitable tissues, including Brugada syndrome (3,4), long QT syndrome (5), chronic pain syndromes (6) and epilepsy (7). Recently, however, VGSCs have been found to have relatively high expression levels in a range of cell types that are considered ‘non-excitable’, including immune cells, fibroblasts and cancer cells (8).

Prostate cancer is the most common type of cancer in males worldwide (9). It has been reported that the expression of certain VGSC α subtypes, such as Nav1.7 (encoded by the SCN9A gene), are upregulated in human and murine prostate cancer cells (10,11). *In vitro* experiments have shown that tetrodotoxin (TTX), a specific VGSC blocker, inhibits cancer invasion, proliferation and migration in PC-3 prostate cancer cells (12,13). Therefore, identifying the aberrant expression of a major ion channel subtype in cancer cells will aid in the targeted diagnosis and treatment of cancer. However, whether the expression of other sodium channel subtypes, other than Nav1.7, is also altered in prostate cancer in humans remains controversial. In addition, whether the expression of sodium channels is altered in individuals with BPH, another common disease of the prostate, compared with that in individuals with a normal prostate or in prostate cancer patients remains unknown. Therefore, the present study set out to determine the mRNA levels of VGSC α subunits in prostate samples from healthy males and BPH patients, and in human prostate cancer cells. By using quantitative polymerase chain reaction (qPCR), we systematically determined the mRNA expression levels of all types of VGSC α subunits in normal human prostate, BPH and prostate cancer cells. By using a patch-clamp technique, it was investigated whether the highly expressed VGSC α subunits in prostate cancer cells were functional. The current study provides a basis for the correlation of VGSC α subunits with prostate cancer and aids in the clinical diagnosis and drug targets for the treatment of prostate cancer.

## Materials and methods

### Tissue samples and cell lines

Three normal human prostate samples and three BPH patient samples were collected from the First Hospital of Shijiazhuang City, China. The age range of the subjects was 50–60 years. Informed consent was obtained from all patients and control subjects who participated in this study. Ethical approval for the study was obtained from the ethics committee of the First Hospital of Shijiazhuang (Shijiazhuang, China). PC-3 and LNCaP cells were purchased from the Cell Bank of the Chinese Academy of Sciences (Shanghai, China).

### Cell culture

PC-3 and LNCaP cells were cultured and harvested as described previously (12,13). In brief, cells were grown in a humidified atmosphere of 5% CO_2_ at 37°C and were maintained in F-12K medium (for PC-3 cells) or in RPMI-1640 medium (for LNCaP cells), with 10% fetal bovine serum (all Gibco-BRL, Carlsbad, CA, USA).

### RNA extraction and purification

Tissue samples were collected, weighed, homogenized and processed for total RNA isolation at 4°C using RNeasy Plus mini kit (Qiagen, Valencia, CA, USA), according to the manufacturer’s instructions. The concentration of total RNA for each sample was determined by the Nanodrop ND-1000 spectrophotometer (Thermo Fisher Scientific, Waltham, MA, USA). The integrity of the extracted RNA was confirmed by electrophoresis under denaturing conditions.

### cDNA synthesis and qPCR

Reverse transcription (RT) was performed using an iScript cDNA synthesis kit (Bio-Rad Laboratories, Inc., Hercules, CA, USA) for the synthesis of single-stranded cDNA library according to the manufacturer’s instructions. qPCR was performed using the iCycler iQ Real-Time PCR detection system (Bio-Rad Laboratories, Inc.), and each sample was run in triplicate. Three controls aimed at detecting DNA contamination in the RNA samples or during the RT or qPCR reactions were always included: i) an RT mixture without reverse transcriptase; ii) an RT mixture including the reverse transcriptase enzyme, but no RNA; and iii) a water only control (reaction mixture with water instead of the cDNA template). Table I lists the primer pairs used for the amplification of each VGSC subtypes and β_2_-microglobulin. PCR products were visualized on a 1.5% agarose gel. The data were collected and analyzed using iCycler software (Bio-Rad Laboratories, Inc.). β_2_-microglobulin was used as internal control. Relative quantification was performed using the comparative threshold (CT) method (ΔΔCT) after determining the CT values for the reference (β_2_-microglobulin) and target (Nav1.1–Nav1.9) genes in each sample set.

### Electrophysiology

Na^+^ currents (*I*_Na_) were recorded using the whole-cell patch-clamp technique as previously described (14–17). In brief, patch pipettes were fabricated from borosilicate glass (Warner Instruments LLC, Hamden, CT, USA) by a P-97 Flaming/Brown micropipette puller (Sutter Instrument, Novato, CA, USA) and fire-polished using a microforge (MF 830; Narishige Scientific Instrument Lab., Tokyo, Japan). Pipette resistance was between 1.0 and 2.0 MΩ, and voltage-clamp experiments were performed with an Axopatch 200B amplifier (Molecular Devices, LLC, Sunnyvale, CA, USA). All recordings were performed at room temperature (20–22°C). *I*_Na_ was recorded in bath solution containing 140 mmol/l NaCl, 1 mmol/l MgCl_2_, 1 mmol/l CaCl_2_, 10 mmol/l HEPES, 3 mmol/l KCl and 10 mmol/l glucose (pH 7.35), adjusted with CsOH. The pipette solution contained the following: 10 mmol/l NaCl, 140 mmol/l CsF, 10 mmol/l EGTA, 5 mmol/l MgATP and 10 mmol/l HEPES (pH 7.35), adjusted with CsOH. Osmolarity was adjusted to 310 mOsm with sucrose for all solutions. Recordings were filtered at 5 kHz and digitally sampled at 40 kHz. To determine the voltage-dependence of steady-state activation, currents were elicited by a 40-msec pulse from a holding potential of −100 mV to test potentials between −80 and +40 mV in 5-mV increments. TTX (Enzo Life Sciences, New York, NY, USA), a VGSC blocker, was used to identify sodium currents.

### Statistical analysis

Statistical analysis was performed using SPSS 17.0 (SPSS, Inc., Chicago, IL, USA). Results are presented as the means ± SEM. Statistical significance of differences between groups was assessed using one-way analysis of variance. P<0.05 was considered to indicate a statistical difference.

## Results

### mRNA expression profile of VGSC α subunits in normal and hyperplastic prostate samples

**Figure 1:**
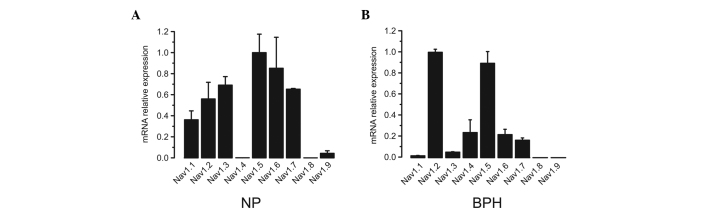
Relative mRNA expression levels of Nav1.1–1.9 α subunits in human normal prostate (NP) and benign prostatic hyperplasia (BPH) samples. Quantitative polymerase chain reaction was used to detect all voltage-gated sodium channel genes in human (A) NP and (B) BPH samples. All data were corrected with β_2_-microglobulin and normalized to Nav1.5 (for NP samples) or to Nav1.2 (for BPH samples). Results were averaged from three different experiments.

Initially, all nine VGSC α subunit expression profiles in normal and hyperplastic prostates were analyzed. Three human normal prostate (NP) and three human BPH biopsy samples were analyzed using qPCR. Isoform-specific primers were used to amplify different subtypes (Table I). In NP samples, with the exception of Nav1.8, all subtypes of VGSC α subunits were detected by qPCR. Among the expressed subtypes, Nav1.2, Nav1.3, Nav1.6, Nav1.7, and particularly Nav1.5, had relatively higher expression levels compared with those of the other subtypes (Fig. 1A). Similar to NP samples, with the exception of Nav1.8 and Nav1.9, all VGSC subtypes were identified in BPH samples (Fig. 1B). Among those subtypes, Nav1.2 and Nav1.5 were the predominant types.

### mRNA expression profile of VGSC α subunits in prostate cancer cells

**Figure 2:**
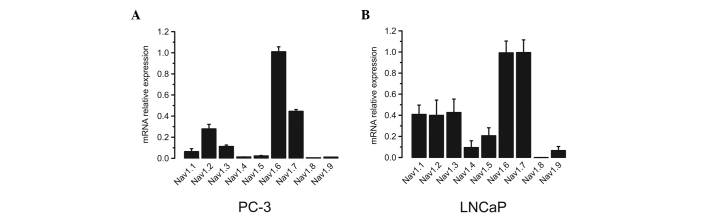
Relative mRNA expression levels of Nav1.1–1.9 α subunits in human prostate cancer cells. Quantitative polymerase chain reaction was used to detect all voltage-gated sodium channel genes in (A) PC-3 and (B) LNCaP cells. All data were corrected with β_2_-microglobulin and normalized to Nav1.6. Results were averaged from three different experiments.

The VGSC α subunit mRNA expression profiles were subsequently determined in human prostate cancer cells. Two typical human prostate cancer cell lines, PC-3 and LNCaP, were utilized. Using the same method as that for normal and hyperplastic prostate samples, it was identified that the expression levels of Nav1.6 were ≥2.5-fold higher than those of any other subtype in PC-3 cells (Fig. 2A). In LNCaP cells, however, Nav1.6 and Nav1.7 were the predominate isoforms, which showed 2- to 3-fold higher expression levels compared with the remaining subtypes (Fig. 2B).

### Nav1.6 and Nav1.7 were up-regulated in prostate cancer cells

**Figure 3:**
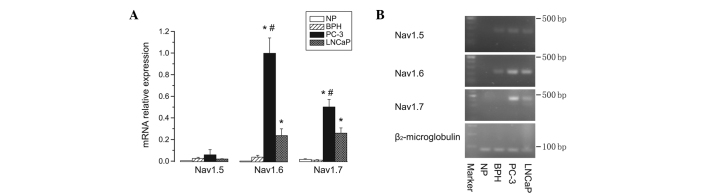
Relative mRNA expression levels of Nav1.5, Nav1.6 and Nav1.7 α subunits in human normal prostate (NP) samples, benign prostatic hyperplasia (BPH) samples and human prostate cancer cells. (A) Real-time quantitative polymerase chain reaction was used to detect all VGSC genes in human normal prostate and BPH samples, as well as PC-3 and LNCaP cell lines. β2-microglobulin was used as a control/reference gene. All data were corrected with β2-microglobulin and normalized to Nav1.6 in PC3 cells. Results were averaged from three different experiments. ^*^P<0.05, compared with NP or BPH samples; ^#^P<0.05, compared with LNCaP cells. (B) Typical qPCR gel images are shown. Marker, 100-bp DNA ladder.

The expression profiles of all VGSC α subunit mRNA levels had previously been determined in each of the three prostate sample types (NP, BPH and prostate cancer cells). To compare the relative expression levels of each VGSC α subunit among all types of prostate cells, Nav1.5, Nav1.6 and Nav1.7 were selected, as these subtypes exhibited the highest expression levels either in NP and BPH samples, or in PC-3 and LNCaP cells, respectively. qPCR data showed that Nav1.5 had almost equally low mRNA expression levels in normal, BPH and prostate cancer cells. Notably, the expression levels of Nav1.6 and Nav1.7, particularly Nav1.6, were significantly upregulated (6- to 27-fold higher) in either PC-3 or LNCaP cancer cells compared with those in NP and BPH samples (P<0.05). Furthermore, the mRNA levels of Nav1.6 and Nav1.7 in PC-3 cells were significantly higher than those in LNCaP cells (P<0.05) (Fig. 3).

### Sodium channels are functional in PC-3 prostate cancer cells

**Figure 4:**
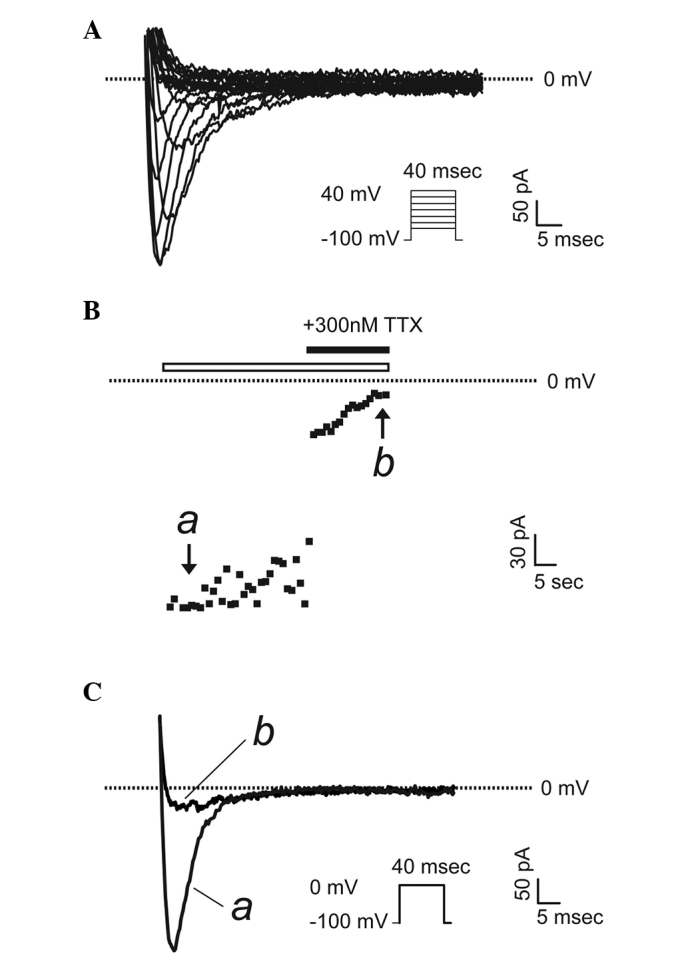
Sodium channels are functional in PC-3 cells. (A) Exemplary Na^+^ channel current traces elicited by 40-msec depolarizing pulses to test potentials between −80 and +40 mV from a holding potential of −100 mV at 5-mV increments. Inset shows a schematic diagram of the voltage-clamp protocol. (B) Time course of tetrodotoxin (TTX; 300 nM) effects on sodium currents recorded by a series of voltage steps from holding potential of −100 mV to a testing pulse at 0 mV is shown. (C) Representative sodium currents at time points ‘a’ and ‘b’ are shown.

To determine whether the VGSC α subunits that were detected to be upregulated in prostate cancer cells were functionally expressed, a patch-clamp technique were used to record whole-cell sodium currents in PC-3 and LNCaP cells, which possess markedly different metastatic potentials. Fig. 4A shows a typical voltage-gated sodium current recorded in PC-3 cells, the more highly metastatic cancer cells, upon a stimulation from a holding potential of -100 mV to a series of test pluses between −80 and +40 mV in 5-mV increments. To confirm the currents were sodium currents, a specific VGSC blocker, TTX (300 nM) was used. The maximum activated currents were completely abolished upon application of TTX (Fig. 4B and C). Notably, in all LNCaP cells (the cells with lower metastatic potential) investigated, there were no sodium currents present (n=15, data not shown).

## Discussion

BPH, a common disease in adult males, has an increasing incidence with age (9). Certain types of prostate cancer are aggressive and have a poor prognosis and high mortality rate in males (18). Previously, it has been reported that certain types of VGSC α subunits were expressed in human and rodent prostate cancer cells (10–13). However, to date, the detailed expression information of VGSC α subunits in the prostate remain unclear. To the best of our knowledge, the current study is the first to use qPCR to explore all VGSC subtype mRNA expression profiles in NP, BPH and prostate cancer cells. The main findings of present study are as follows: i) the majority of VGSC α subunits can be detected in NP and BPH samples; however, Nav1.6/Nav1.7 in NP and BPH samples exhibit very low expression levels compared with those in prostate cancer cells; ii) in prostate cancer (PC-3 and LNCaP) cells, the expression of Nav1.6 and Nav1.7 is dramatically upregulated; and iii) the upregulated Nav1.6 and Nav1.7 α subunits in PC-3 cancer cells are functional.

Ion channels are a class of important functional membrane-spanning proteins responsible for ion permission and ion carrying. VGSCs are key ion channels to generate and conduct action potential in excitable tissues (such as nerve and muscle) (2). VGSCs are composed of a pore-forming α subunit and are associated with one or more auxiliary subunits (β1–β4). Nine sodium channel α subunits (Nav1.1-Nav1.9), encoded by the SCN1A-SCN5A and SCN8A-SCN11A genes, have been found in vertebrates. The α subunits consist of four homologous domains, and each domain contains six transmembrane segments (S1–S6), wherein a positively charged S4 acts as a voltage sensor. β subunits are regulatory subunits responsible for channel gating and trafficking (2).

Increasing evidence shows that VGSCs also exist in non-excitatory tissues, such as glial cells, osteoblasts, lymphocytes, endothelial cells and fibroblasts (19–22). A series of studies have found VGSCs expressed in prostate cancer cells, and the expression of VGSCs were positively correlated with cancer proliferation (12,13,23–25). VGSCs were identified to be specifically expressed in the MAT-LyLu highly metastatic murine prostate cancer cell line and, using TTX blocking, VGSCs can significantly reduce the invasiveness of the cell line *in vitro* (13), suggesting that VGSC expression is important in the invasion and metastasis of cancer cells. Further study found that the expression levels of Nav1.7 are 3-fold higher in highly metastatic prostate cancer cell lines (MAT-LyLu and PC-3) than in weakly metastatic cell lines (AT-2 and LNCaP) (23). The present study demonstrated that not only Nav1.7 but also Nav1.6 mRNA levels were significantly increased in prostate cancer cell lines. Notably, Nav1.6 and Nav1.7 mRNA are expressed at higher levels in PC-3 cells than in LNCaP cells. PC-3 and LNCaP cells are two different metastatic prostate cancer cell lines, and PC-3 cells have a greater invasive capacity than LNCaP cells. The functional testing in the current study also showed typical sodium currents are only present in PC-3 cells. These findings suggest that inhibition of VGSC α subunits (Nav1.6 and Nav1.7) may be a useful treatment strategy to reduce the metastatic spread of prostate cancer. To clarify whether the upregulation of VGSC α subunits is disease-associated, we also detected and compared the VGSC α subunit expression profiles in prostate biopsy samples from normal subjects and from BPH patients. The majority of VGSC subtypes were expressed basically at very low levels in those samples.

As for the mechanisms accounting for sodium channels affecting cancer cell migration and metabolism, there are several different interpretations. Brisson *et al* propose that in MDA-MB-231 breast cancer cells, Nav1.5 increases Na^+^ influx, which activates the Na^+^/H^+^ exchanger type 1, which is an important regulator of H^+^ efflux. Increased H^+^ release alters the pH of the local pH, leading to pH-dependent extracellular matrix degradation and cell invasiveness (26). However, Carrithers *et al* suggests that Nav1.6 regulates cellular invasion through its effects on podosome and invadopodia formation in macrophages and melanoma cells (27). Overall, the mechanisms by which sodium channel mRNA is upregulated and sodium channels regulate cell invasion in cancer cells require further study.

Clinical pathological staging, biopsy Gleason score and prostate-specific antigen have been widely used in the diagnosis and monitoring of disease progression of prostate cancer (28,29). However, due to the low specificity and sensitivity, the application is limited. With the progress of high-throughput genomics and proteomics technology, increasing prostate cancer-related tumor markers, such as MIB-1/Ki67 labeling indices, DD3, EGR-1 and Bcl-2, are constantly being discovered and used in the diagnosis and prediction of prognosis of prostate cancer (30,31). However, thus far, there remains a lack of specific and sensitive tumor markers that can accurately diagnose early prostate cancer and determine its invasiveness. Therefore, it has become important to study new molecular biomarker of genotype and phenotype in prostate cancer cells. Previous studies have demonstrated that sodium channels influence the metabolism of cancer cells through affecting cancer cell adhesion, proliferation, invasion, migration and apoptosis (8,10–13), suggesting that sodium channels may be an important diagnosis indicator for prostate cancer. The present study showed that mRNA expression levels of Nav1.6 and Nav1.7 were significantly upregulated in prostate cancer cells compared with those in NP or BPH samples, suggesting Nav1.6 and Nav1.7 may be potential diagnostic markers for prostate cancer. It has been reported that prostate surgery using classical sodium channel blockers/anesthetic drugs could inhibit prostate cancer spread and recurrence (32). Therefore, it may be speculated that sodium channel blockers, particularly Nav1.6 and Nav1.7 α subunit-specific blockers, have the potential to participate in the prevention and treatment of prostate cancer.

Overall, VGSCs have been demonstrated to be upregulated in numerous types of metastatic prostate cancer cells. The upregulated sodium channels play important roles in regulating cellular migration, invasion and proliferation. Notably, the altered VGSC expression has a potential utility as a diagnostic and therapeutic target. In the present study, the mRNA expression levels of all nine types of VGSCs α subunit were analyzed in human NP, BPH and prostate cancer (PC-3 and LNCaP) cells by qPCR assay. Compared with those in NP and BPH samples, the mRNA expression levels of Nav1.6 and Nav1.7 were dramatically upregulated in prostate cancer cells, suggesting these subtypes may be potential diagnostic markers for certain types of prostate cancer in humans.

## Figures and Tables

**Table I:** qPCR primer pairs used for detecting VGSC α subunits mRNA levels in human normal prostate, in BPH samples and in human prostate cancer cells.

Gene symbol (human)	Channel name	Forward primer (5′-3′)	Reverse primer (5′-3′)	Product length (bp)
SCN1A	Nav1.1	CAGTGCAGCAGGCAGGC	TCAATCGGTTCCCTTCAATGGAG	212
SCN2A	Nav1.2	AGACTTCAGTGGTGCTGGTG	CTCTTCTTCTCCAGACTGTTC	139
SCN3A	Nav1.3	GGGTTAGGAGAGCTGTTGG	CAAGGTGCTCTCTCTGTCTTC	109
SCN4A	Nav1.4	CTCGAGCTGGACCACCTTAA	TCTCCTCTGCCTGCTCCTC	232
SCN5A	Nav1.5	CAACAGCTGGAATATCTTCG	CCAAAGATGGAGTAGATGAAC	260
SCN8A	Nav1.6	TCAGCATCCCAGGCTCGC	CTGGCTGTAGCCGCTGTA	223
SCN9A	Nav1.7	TATGACCATGAATAACCC	TCAGGTTTCCCATGAACAGC	389 (297^a^)
SCN10A	Nav1.8	GTTGGCACAGCAATAGATCTCC	GACAGCCATGTCATTCTTGAC	246
SCN11A	Nav1.9	CCATCCTTGACCATCTCAACTG	GGAAAGGAATGTGCTCCTGA	186
β2-microglobulin		TGCTGTCTCCATGTTTGATGTATCT	TCTCTGCTCCCCACCTCTAAGT	80

a qPCR product length of channel splice variant.

## References

[1] Catterall WA (1984). The molecular basis of neuronal excitability. Science.

[2] Catterall WA (2000). From ionic currents to molecular mechanisms: the structure and function of voltage-gated sodium channels. Neuron.

[3] Antzelevitch C, Brugada P, Borggrefe M (2005). Brugada syndrome: report of the second consensus conference: endorsed by the Heart Rhythm Society and the European Heart Rhythm Association. Circulation.

[4] Veltmann C, Schimpf R, Echternach C (2006). A prospective study on spontaneous fluctuations between diagnostic and non-diagnostic ECGs in Brugada syndrome: implications for correct phenotyping and risk stratification. Eur Heart J.

[5] Bennett PB, Yazawa K, Makita N, George AL (1995). Molecular mechanism for an inherited cardiac arrhythmia. Nature.

[6] Waxman SG Painful Na-channelopathies: an expanding universe. Trends Mol Med.

[7] Oliva M, Berkovic SF, Petrou S (2012). Sodium channels and the neurobiology of epilepsy. Epilepsia.

[8] Brackenbury WJ (2012). Voltage-gated sodium channels and metastatic disease. Channels (Austin).

[9] Baade PD, Youlden DR, Krnjacki LJ (2009). International epidemiology of prostate cancer: geographical distribution and secular trends. Mol Nutr Food Res.

[10] Diss JK, Stewart D, Pani F (2005). A potential novel marker for human prostate cancer: voltage-gated sodium channel expression in vivo. Prostate Cancer Prostatic Dis.

[11] Diss JK, Fraser SP, Walker MM, Patel A, Latchman DS, Djamgoz MB (2008). Beta-subunits of voltage-gated sodium channels in human prostate cancer: quantitative in vitro and in vivo analyses of mRNA expression. Prostate Cancer Prostatic Dis.

[12] Laniado ME, Lalani EN, Fraser SP (1997). Expression and functional analysis of voltage-activated Na^+^ channels in human prostate cancer cell lines and their contribution to invasion in vitro. Am J Pathol.

[13] Grimes JA, Fraser SP, Stephens GJ (1995). Differential expression of voltage-activated Na^+^ currents in two prostatic tumour cell lines: contribution to invasiveness in vitro. FEBS Lett.

[14] Nguyen TP, Wang DW, Rhodes TH, George AL (2008). Divergent biophysical defects caused by mutant sodium channels in dilated cardiomyopathy with arrhythmia. Circ Res.

[15] Benson DW, Wang DW, Dyment M (2003). Congenital sick sinus syndrome caused by recessive mutations in the cardiac sodium channel gene (SCN5A). J Clin Invest.

[16] Sato PY, Musa H, Coombs W (2009). Loss of plakophilin-2 expression leads to decreased sodium current and slower conduction velocity in cultured cardiac myocytes. Circ Res.

[17] Wang C, Hennessey JA, Kirkton RD (2011). Fibroblast growth factor homologous factor 13 regulates Na^+^ channels and conduction velocity in murine hearts. Circ Res.

[18] Nomiya T, Tsuji H, Toyama S (2013). Management of high-risk prostate cancer: radiation therapy and hormonal therapy. Cancer Treat Rev.

[19] Diaz D, Delgadillo DM, Hernández-Gallegos E (2007). Functional expression of voltage-gated sodium channels in primary cultures of human cervical cancer. J Cell Physiol.

[20] Fraser SP, Diss JK, Chioni AM (2005). Voltage-gated sodium channel expression and potentiation of human breast cancer metastasis. Clin Cancer Res.

[21] Hernandez-Plata E, Ortiz CS, Marquina-Castillo B (2012). Overexpression of NaV 1.6 channels is associated with the invasion capacity of human cervical cancer. Int J Cancer.

[22] Roger S, Besson P, Le Guennec JY (2003). Involvement of a novel fast inward sodium current in the invasion capacity of a breast cancer cell line. Biochim Biophys Acta.

[23] Fraser SP, Grimes JA, Djamgoz MB (2000). Effects of voltage-gated ion channel modulators on rat prostatic cancer cell proliferation: comparison of strongly and weakly metastatic cell lines. Prostate.

[24] Abdul M, Hoosein N (2002). Voltage-gated sodium ion channels in prostate cancer: expression and activity. Anticancer Res.

[25] Smith P, Rhodes NP, Shortland AP (1998). Sodium channel protein expression enhances the invasiveness of rat and human prostate cancer cells. FEBS Lett.

[26] Brisson L, Gillet L, Calaghan S (2011). Na(V)1.5 enhances breast cancer cell invasiveness by increasing NHE1-dependent H(+) efflux in caveolae. Oncogene.

[27] Carrithers MD, Chatterjee G, Carrithers LM (2009). Regulation of podosome formation in macrophages by a splice variant of the sodium channel SCN8A. J Biol Chem.

[28] Diamandis EP, Yu H (1997). Nonprostatic sources of prostate-specific antigen. Urol Clin North Am.

[29] Foster CS, Cornford P, Forsyth L, Djamgoz MB, Ke Y (1999). The cellular and molecular basis of prostate cancer. BJU Int.

[30] de Kok JB, Verhaegh GW, Roelofs RW (2002). DD3 (PCA3), a very sensitive and specific marker to detect prostate tumors. Cancer Res.

[31] Chakravarti A, Zhai GG (2003). Molecular and genetic prognostic factors of prostate cancer. World J Urol.

[32] Biki B, Mascha E, Moriarty DC, Fitzpatrick JM, Sessler DI, Buggy DJ (2008). Anesthetic technique for radical prostatectomy surgery affects cancer recurrence: a retrospective analysis. Anesthesiology.

